# Invasive *Conidiobolus* infection in an immunocompromised pediatric patient in Southern California

**DOI:** 10.1128/asmcr.00155-25

**Published:** 2025-11-18

**Authors:** Jacky Lu, Samuel M. Goodfellow, Esther Vaugon, Andrew Karnaze, Cristina Costales, Michael A. Smit, Jennifer Dien Bard

**Affiliations:** 1 Department of Pathology and Laboratory Medicine, Children’s Hospital Los Angeles 635205, Los Angeles, California, USA; 2 Department of Laboratory Medicine, CHU Sainte-Justine, University of Montreal 5622, Montreal, Québec, Canada; 3 Department of Pediatrics, Division of Infectious Diseases, Children’s Hospital Los Angeles 337885, Los Angeles, California, USA; 4 Keck School of Medicine, University of Southern California 12223, Los Angeles, California, USA; Rush University Medical Center, Chicago, Illinois, USA

**Keywords:** *Conidiobolus*, plasma cell-free DNA sequencing, invasive fungal infection

## Abstract

**Background:**

*Conidiobolus* species are largely known to cause rhinofacial cellulitis in healthy individuals but can manifest as an invasive infection in immunocompromised patients. Few cases have been described in the literature as they are difficult to diagnose and rarely observed in North America, particularly in the pediatric population.

**Case Summary:**

A 15-year-old boy with a history of multiple bone marrow transplants for mixed phenotype acute leukemia presented to the emergency department with febrile neutropenia and chills. His clinical course was complicated by potential *Rothia mucilaginosa* bacteremia, but imaging revealed a potential invasive pulmonary fungal infection. Following rapid decompensation and admission in the intensive care unit, plasma cell-free DNA sequencing was ordered, identifying *Conidiobolus* species. The same organism was then isolated in the patient’s sputum culture shortly thereafter. This organism was found to demonstrate high minimum inhibitory concentration to antifungals tested *in vitro*. Ultimately, the patient was discharged on posaconazole and trimethoprim-sulfamethoxazole.

**Conclusion:**

*Conidiobolus* can be an agent of invasive fungal infection and may be considered a potential infectious agent in the immunocompromised patient. Treatment and outcomes of invasive *Conidiobolus* remain elusive.

## INTRODUCTION

*Conidiobolus* belongs to the *Entomophthorales* group and is closely related to the *Mucorales* group (1). Historically, these groups both belonged to the phylum *Zygomycota*. Following a comprehensive phylogenetic study based on multigene sequence analysis, the order *Mucorales* now falls under the subphyla of *Mucoromycotina*, while the order *Entomophthorales* is part of the *Entomophthoromycotina* subphyla (2). Whereas the *Mucorales* group is known to cause invasive disease in immunocompromised patients and through direct inoculation during trauma, *Conidiobolus* species are a known cause of conidiobolomycosis. The most common manifestation is rhinofacial cellulitis, which may lead to chronic facial deformity in immunocompetent hosts (3–5). However, few cases of *Conidiobolus* invasive infection have been described in the literature, infecting other viscera or the central nervous system and often involving multiple organs (6). Due to their growth requirements of high-level humidity, it has been postulated that pathogenicity may be limited to tropical or subtropical regions (4). Diagnosis generally relies on histological examination of biopsied or resected tissue, as cultures show poor recovery of the causative organism, negative in more than 85% of cases (7). Similar to fungal species in the *Mucorales* group, the fungal antigen (1,3)-beta-D-glucan test is often negative in *Entomophthorales*, despite the presence of significant amounts of (1,3)-D-beta-glucan in the cell wall (4), although most cases are localized. Detection of (1,3)-beta-D-glucan in patients with disseminated *Conidiobolus* infections has been inconsistent and may be species dependent (1, 8). Here, we describe a rare case of invasive *Conidiobolus* infection in an immunocompromised patient in Southern California.

## CASE PRESENTATION

A 15-year-old male presented to the emergency department (ED) with chills and fever (maximum temperature: 39.6°C) in the setting of neutropenia following chemotherapy at an urban academic, pediatric quaternary-care center in Southern California. The patient had received his third bone marrow transplantation (matched unrelated donor peripheral blood stem cell) 20 months prior to hospital admission with leukemic relapse 11 months later and was receiving palliative chemotherapy with azacitidine, venetoclax, and gemtuzumab for active leukemic disease. He has had a history of graft-versus-host disease (GVHD) of the skin (stage 3) after completion of GVHD prophylaxis, 53 days post-transplant, which had since resolved. On admission to the oncology unit, at 9 months after his most recent relapse, he had an absolute neutrophil count of 0.00 and an elevated C-reactive protein level of 22.9 mg/dL (normal level: <0.9 mg/dL). A single blood culture set (aerobic and anaerobic bottles) was collected in the ED, and *Rothia mucilaginosa* was recovered from a single aerobic blood culture bottle after 13.5 hours of incubation. While the significance of this culture result was unclear, he was started on vancomycin (500 mg, IV intermittent, Q8H) and maintained on cefepime (1,500 mg, IV, Q8H) for broad gram-negative coverage. Prior to hospital admission, he was receiving prophylactic trimethoprim-sulfamethoxazole (400 mg–80 mg oral tablet, PO, BID SaSu) and levofloxacin (250 mg, PO, Q24H). Vancomycin was discontinued after 6 days as the patient showed improvement, and subsequent blood cultures were negative.

Due to his prolonged febrile neutropenia despite broad-spectrum antibiotics, CT scans of the chest, abdomen, and pelvis with contrast were performed, which showed signs of left lower lobe pneumonia. The patient was on room air and not short of breath or tachypneic but was complaining of left flank pain. There was concern that the etiology of the pneumonia could have been *R. mucilaginosa* (9), given prior blood culture results, versus a fungal etiology. Of note, the patient had already been on therapeutic dosing of isavuconazonium (372 mg, PO, Q24H) for treatment of pulmonary aspergillosis diagnosed 9 months earlier. Therefore, there was concern for the development of resistant pulmonary aspergillosis, given reports of poor compliance with isavuconazonium, versus a new fungal pneumonia. Both serum (1,3)-beta-D-glucan (reference range: <31 pg/mL) and *Aspergillus* galactomannan were negative (reference range: <0.5 optical density index), and the patient remained stable with no progression of pulmonary symptoms.

However, 2 days after his CT scans, he was transferred to the pediatric intensive care unit (PICU) for hypotension secondary to a suspected gastrointestinal bleed. Due to his prolonged febrile neutropenia despite broad-spectrum antimicrobials, imaging suggestive of invasive fungal infection, and inability to collect bronchioloalveolar lavage (BAL) due to clinical instability, plasma cell-free DNA metagenomic next-generation sequencing (mNGS) testing (Karius Spectrum, Redwood City, CA, USA) was approved by the medical microbiologist. At the time of plasma collection, the patient was empirically treated with isavuconazonium, with proper compliance for at least 13 days since admission. A positive report for *Conidiobolus incongruus* (2,327 molecules per 100 nL) was received 2 days after collection (day 16 of admission).

Based on previous case reports and the variable resistance of *C. incongruus*, its detection prompted initiation of liposomal amphotericin B (150 mg, IV, QDay) and IV trimethoprim-sulfamethoxazole ([80 mg–16 mg/mL IV solution] 160 mg, IV, Q8H) and evaluation by otolaryngology. Nasal endoscopic exam was normal, and CT scan of the sinuses did not reveal evidence of rhinosinusitis. Due to his recent GI bleed and ongoing thrombocytopenia, a biopsy was not recommended for the patient’s safety. Ultrasound of the chest showed a large left pleural effusion.

**Fig 1:**
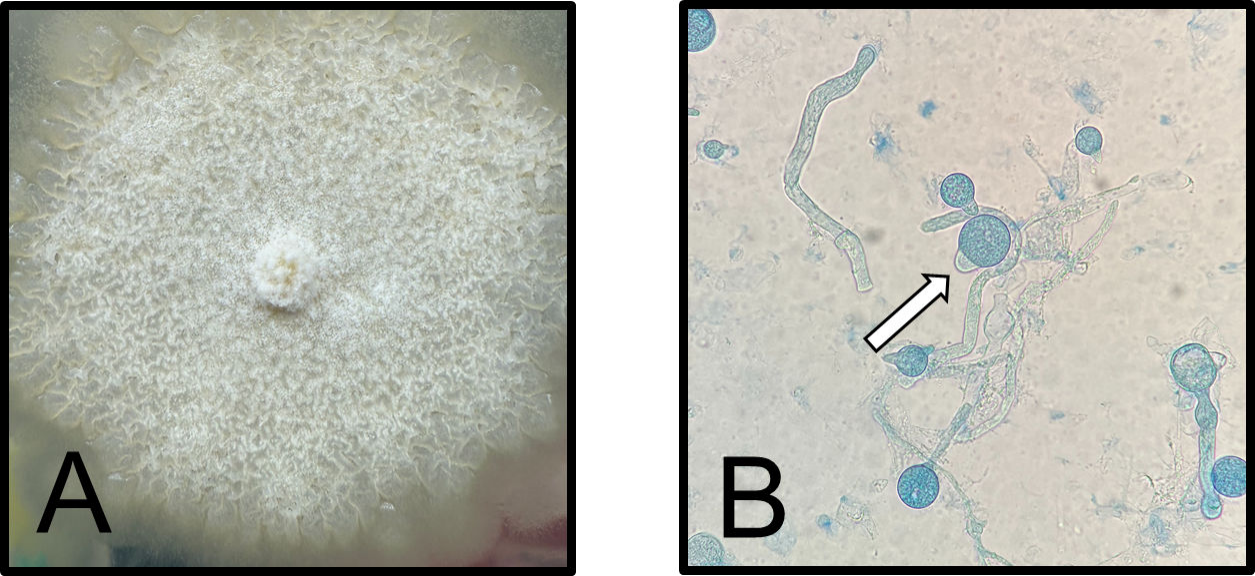
*Conidiobolus* spp. isolated from sputum. (**A**) Macroscopic examination of mold grown on Sabouraud dextrose agar after 3 days at 30°C. (**B**) Lactophenol blue preparation of mold examined at ×40. Conidia with prominent papillae were observed (arrow).

A sputum sample was successfully collected on day 16 of hospitalization (day 3 in PICU) and submitted to the microbiology laboratory for bacterial and fungal cultures. The Gram stain revealed few white blood cells, few squamous epithelial cells, and no organisms seen. Mold was recovered from chocolate agar 48 hours after incubation at 37°C in 5% CO_2_, along with normal respiratory flora. Following subculture to Sabouraud dextrose agar, the mold was macroscopically described as flat, waxy, and buff colored with white aerial hyphae after 3 days of incubation at 30°C in ambient air (Fig. 1A). Microscopic examination with lactophenol blue revealed unbranched non-septate hyphae resembling the Mucorales group. Conidia with prominent papillae were also observed (Fig. 1B). The mold was reported as *Conidiobolus* spp. solely based on microscopic morphology. Testing by MALDI-TOF MS (MALDI Biotyper, Bruker) was attempted but was unsuccessful in yielding sufficient spectra score for pathogen identification, highlighting the difficulty of identifying uncommon molds by this method. The isolate was sent to the University of Texas Health, South Texas Reference Laboratories in San Antonio for antifungal susceptibility testing and confirmation by ITS sequencing, which identified the organism to the genus level. *In vitro* susceptibility testing revealed the following minimum inhibitory concentration (MIC): amphotericin B (MIC, 1 mcg/mL), itraconazole (MIC, >16 mcg/mL), posaconazole (MIC, >16 mcg/mL), and isavuconazole (MIC, >16 mcg/mL). There are no available clinical breakpoints from the Clinical and Laboratory Standards Institute (CLSI) or European Committee on Antimicrobial Susceptibility Testing (EUCAST).

Given the prognosis of invasive fungal infection, a multidisciplinary care team made the decision to de-escalate harmful medical interventions, including IV liposomal amphotericin B, and focus on comfort care. The patient was ultimately discharged on trimethoprim-sulfamethoxazole (160 mg PO, TID), posaconazole (300 mg PO, QD), and prophylactic levofloxacin (250 mg PO, QD) on day 26 of hospital admission. The patient is being followed by the outpatient clinic.

## DISCUSSION

There have been only 10 cases of invasive *Conidiobolu*s spp. (including *C. incongruus*, *C. coronatus*, and *C. lamprauges*) infection described in the literature since 2020 (6). However, the true number of invasive cases is likely underreported as this organism is notoriously difficult to recover by culture, and infections largely occur in underresourced regions (4). Of the 10 invasive cases reviewed by Stavropoulou et al., which encompasses all the cases since the one we are reporting, 7 had multiorgan with pulmonary involvement (8, 10–16). The prognosis of invasive disease caused by *Conidiobolu*s spp. is poor, with 7 of the 10 invasive cases being fatal (1, 8, 12–15, 17), 3 of which were in children (10, 16, 17). The majority of these patients had some form of immunosuppression (1, 6, 8, 14–17), and five were patients in the United States (10, 13–17). There is no consensus for treatment, although a high level of *in vitro* azole resistance has been reported (18, 19). Treatment for both invasive and chronic rhinofacial diseases has included combinations of azoles, amphotericin B, trimethoprim-sulfamethoxazole, potassium iodide, terbinafine, hyperbaric oxygen, and surgical debridement, with varying success (6, 15, 20). Our patient was prescribed IV liposomal amphotericin B and trimethoprim-sulfamethoxazole, but due to progression of the oncological disease, the decision was made to proceed with comfort care, and the patient was thus transitioned to broad-spectrum oral azole.

*Conidiobolus* is found in the soil and water, and therefore the significance of *Conidiobolus* detection from plasma cell-free DNA mNGS was unclear, as potential laboratory contamination could not be ruled out. Recovery of the organism from the patient’s sputum sample collected 3 days later supported its significance as the cause of the patient’s pulmonary disease. Detection of cell-free DNA in the plasma may be representative of angioinvasion, but a tissue biopsy would be required to confirm this. Interestingly, this organism is not generally known to be angioinvasive (5, 21).

There are conflicting reports on the utility of plasma cell-free DNA mNGS to aid in the diagnosis of infections, including invasive fungal infections (22–24). In our institution, the test may be considered in patients where direct source specimens of infected tissue or viscera, such as BAL, are unattainable. In this case, the test was deemed appropriate for the patient as invasive fungal infection was highly suspected, and a BAL collection was deemed high risk. The speed of the test is noteworthy, as a result was provided within 2 days from specimen collection. It is important to also note that sputum culture was successful at recovering the pathogen within 2 days of incubation as well, highlighting that conventional, standard of care testing is often sufficient in many cases if appropriate, high-quality specimens can be collected.

### Conclusions

Here we describe a rare case of invasive pulmonary disease caused by *Conidiobolus* species in an immunocompromised adolescent. A combination of traditional culture and molecular approaches aided in the diagnosis of this infection. Invasive disease caused by *Conidiobolus* is likely underestimated due to limitations in recovery by culture; treatment and outcome remain inconsistent.

## References

[1] Wüppenhorst N, Lee M-K, Rappold E, Kayser G, Beckervordersandforth J, de With K, Serr A. 2010. Rhino-orbitocerebral zygomycosis caused by Conidiobolus incongruus in an immunocompromised patient in Germany. J Clin Microbiol 48:4322–4325. doi:10.1128/JCM.01188-10 20861341 PMC3020825

[2] Hibbett DS, Binder M, Bischoff JF, Blackwell M, Cannon PF, Eriksson OE, Huhndorf S, James T, Kirk PM, Lücking R, et al.. 2007. A higher-level phylogenetic classification of the Fungi. Mycol Res 111:509–547. doi:10.1016/j.mycres.2007.03.004 17572334

[3] Shaikh N, Hussain KA, Petraitiene R, Schuetz AN, Walsh TJ. 2016. Entomophthoramycosis: a neglected tropical mycosis. Clin Microbiol Infect 22:688–694. doi:10.1016/j.cmi.2016.04.005 27109491

[4] Vilela R, Mendoza L. 2018. Human pathogenic entomophthorales. Clin Microbiol Rev 31:e00014-18. doi:10.1128/CMR.00014-18 30158298 PMC6148186

[5] Prabhu RM, Patel R. 2004. Mucormycosis and entomophthoramycosis: a review of the clinical manifestations, diagnosis and treatment. Clin Microbiol Infect 10:31–47. doi:10.1111/j.1470-9465.2004.00843.x 14748801

[6] Stavropoulou E, Coste AT, Beigelman-Aubry C, Letovanec I, Spertini O, Lovis A, Krueger T, Burger R, Bochud PY, Lamoth F. 2020. Conidiobolus pachyzygosporus invasive pulmonary infection in a patient with acute myeloid leukemia: case report and review of the literature. BMC Infect Dis 20:527. doi:10.1186/s12879-020-05218-w 32698804 PMC7374966

[7] Hernandez MJ, Landaeta W, Salazar BN, Vargas J, Rodriguez-Morales AJ. 2007. Subcutaneous zygomycosis due to Conidiobolus incongruus. Int J Infect Dis 11:468–470. doi:10.1016/j.ijid.2007.01.001 17331786

[8] Kimura M, Yaguchi T, Sutton DA, Fothergill AW, Thompson EH, Wickes BL. 2011. Disseminated human conidiobolomycosis due to Conidiobolus lamprauges. J Clin Microbiol 49:752–756. doi:10.1128/JCM.01484-10 21147951 PMC3043483

[9] Maraki S, Papadakis IS. 2015. Rothia mucilaginosa pneumonia: a literature review. Infect Dis (Lond) 47:125–129. doi:10.3109/00365548.2014.980843 25664502

[10] Gilbert EF, Khoury GH, Pore RS. 1970. Histopathological identification of Entomophthora phycomycosis. Deep mycotic infection in an infant. Arch Pathol 90:583–587. 5485116

[11] King DS, Jong SC. 1976. Identity of the etiological agent of the first deep entomophthoraceous infection of man in the United States. Mycologia 68:181–183. doi:10.2307/3758911 945455

[12] Busapakum R, Youngchaiyud U, Sriumpai S, Segretain G, Fromentin H. 1983. Disseminated infection with Conidiobolus incongruus. Med Mycol 21:323–330. doi:10.1080/00362178385380471 6686345

[13] Jaffey PB, Haque AK, el-Zaatari M, Pasarell L, McGinnis MR. 1990. Disseminated Conidiobolus infection with endocarditis in a cocaine abuser. Arch Pathol Lab Med 114:1276–1278. 2252425

[14] Walker SD, Clark RV, King CT, Humphries JE, Lytle LS, Butkus DE. 1992. Fatal disseminated Conidiobolus coronatus infection in a renal transplant patient. Am J Clin Pathol 98:559–564. doi:10.1093/ajcp/98.6.559 1334363

[15] Walsh TJ, Renshaw G, Andrews J, Kwon-Chung J, Cunnion RC, Pass HI, Taubenberger J, Wilson W, Pizzo PA. 1994. Invasive zygomycosis due to Conidiobolus incongruus. Clin Infect Dis 19:423–430. doi:10.1093/clinids/19.3.423 7811860

[16] Erker C, Huppler AR, Walsh TJ, McCormick ME, Suchi M, Bhatt NS, Kehl SC, Southwood J, Harker-Murray P. 2018. Successful treatment of invasive Conidiobolus infection during therapy for acute lymphoblastic leukemia. J Pediatr Hematol Oncol 40:e446–e449. doi:10.1097/MPH.0000000000000985 28991126 PMC5904005

[17] Radhakrishnan N, Sachdeva A, Oberoi J, Yadav SP. 2009. Conidiobolomycosis in relapsed acute lymphoblastic leukemia. Pediatr Blood Cancer 53:1321–1323. doi:10.1002/pbc.22259 19731329

[18] Guarro J, Aguilar C, Pujol I. 1999. In-vitro antifungal susceptibilities of Basidiobolus and Conidiobolus spp. strains. J Antimicrob Chemother 44:557–560. doi:10.1093/jac/44.4.557 10588321

[19] Tondolo JSM, Loreto ES, Jesus FPK, Dutra V, Nakazato L, Alves SH, Santurio JM. 2018. In vitro assessment of antifungal drugs and sulfamethoxazole-trimethoprim against clinical isolates of Conidiobolus lamprauges. Antimicrob Agents Chemother 62:e01685-17. doi:10.1128/AAC.01685-17 29439970 PMC5913962

[20] Sharath S, Bansal A, Sinha S, Ahuja A. 2025. Successful management of rhinofacial Conidiobolus coronatus infection with itraconazole monotherapy. Am J Trop Med Hyg 112:158–160. doi:10.4269/ajtmh.24-0196 39471509 PMC11720784

[21] Ribes JA, Vanover-Sams CL, Baker DJ. 2000. Zygomycetes in human disease. Clin Microbiol Rev 13:236–301. doi:10.1128/CMR.13.2.236 10756000 PMC100153

[22] Kaur I, Shaw B, Multani A, Pham C, Malhotra S, Smith E, Adachi K, Allyn P, Bango Z, Beaird OE, et al.. 2025. Real-world clinical impact of plasma cell-free DNA metagenomic next-generation sequencing assay. Infect Control Hosp Epidemiol 46:1–8. doi:10.1017/ice.2024.242 PMC12034450 39881594

[23] Vissichelli NC, Morales MK, Kolipakkam B, Bryson A, Sabo RT, Toor AA. 2023. Cell-free next-generation sequencing impacts diagnosis and antimicrobial therapy in immunocompromised hosts: a retrospective study. Transpl Infect Dis 25:e13954. doi:10.1111/tid.13954 36632004

[24] Huygens S, Schauwvlieghe A, Wlazlo N, Moors I, Boelens J, Reynders M, Chong GL, Klaassen CHW, Rijnders BJA. 2024. Diagnostic value of microbial cell-free DNA sequencing for suspected invasive fungal infections: a retrospective multicenter cohort study. Open Forum Infect Dis 11:ofae252. doi:10.1093/ofid/ofae252 38868302 PMC11166502

